# Bridging The Age Gap: observational cohort study of effects of chemotherapy and trastuzumab on recurrence, survival and quality of life in older women with early breast cancer

**DOI:** 10.1038/s41416-021-01388-9

**Published:** 2021-05-10

**Authors:** Alistair Ring, Nicolò Matteo Luca Battisti, Malcolm W. R. Reed, Esther Herbert, Jenna L. Morgan, Michael Bradburn, Stephen J. Walters, Karen A. Collins, Sue E. Ward, Geoffrey R. Holmes, Maria Burton, Kate Lifford, Adrian Edwards, Thompson G. Robinson, Charlene Martin, Tim Chater, Kirsty J. Pemberton, Alan Brennan, Kwok Leung Cheung, Annaliza Todd, Riccardo A. Audisio, Juliet Wright, Richard Simcock, Tracey Green, Deirdre Revell, Jacqui Gath, Kieran Horgan, Chris Holcombe, Matthew C. Winter, Jay Naik, Rishi Parmeshwar, Margot A. Gosney, Matthew Q. Hatton, Alastair M. Thompson, Lynda Wyld, on behalf of the Age Gap TMG, Karen Collins, Sue Ward, Geoff Holmes, Jenna Morgan, Mike Bradburn, Stephen Walters, Maria Burton, Kate Lifford, Adrian Edwards, Kate Brain, Alistair Ring, Thomson Robinson, Kirsty Pemberton, Anne Shrestha, Anthony Nettleship, Paul Richards, Kwok Leung Cheung, Helena Harder, Riccardo Audisio, Nicolò Matteo Luca Battisti, Juliette Wright, Richard Simcock, Chris Murray, Alistair M. Thomson, Margot Gosney, Matthew Hatton, Fiona Armitage, Julietta Patnick, Tracy Green, Deirdre Revill, Jacqui Gath, Kieran Horgan, Chris Holcombe, Matt Winter

**Affiliations:** 1 Department of Medicine, Breast Unit, The Royal Marsden Hospital NHS Foundation Trust, London, UK & Breast Cancer Research Division, The Institute of Cancer Research, London, UK; 2 Brighton and Sussex Medical School, Brighton, UK; 3 Clinical Trials Research Unit, School for Health and Related Research, University of Sheffield, Sheffield, UK; 4 Department of Oncology and Metabolism, University of Sheffield Medical School, Sheffield, UK; 5 College of Health, Wellbeing and Life Sciences, Department of Allied Health Professions, Sheffield Hallam University, Sheffield, UK; 6 Department of Health Economics and Decision Science, School for Health and Related Research (ScHARR), University of Sheffield, Sheffield, UK; 7 Division of Population Medicine, Cardiff University, Cardiff, UK; 8 Department of Cardiovascular Sciences and NIHR Biomedical Research Centre, University of Leicester, Cardiovascular Research Centre, Leicester, UK; 9 School of Medicine, University of Nottingham, Royal Derby Hospital, Derby, UK; 10 University of Gothenberg, Sahlgrenska Universitetssjukhuset, Göteborg, Sweden; 11 Sussex Cancer Centre, Royal Sussex County Hospital, Brighton, UK; 12 Yorkshire and Humber Consumer Research Panel, Cottingham, UK; 13 Department of Breast Surgery, Bexley Cancer Centre, St James’s University Hospital, Leeds, UK; 14 Liverpool University Hospitals Foundation Trust, Liverpool, UK; 15 Weston Park Hospital, Sheffield, UK; 16 Pinderfields Hospital, Mid Yorkshire NHS Foundation Trust, Wakefield, UK; 17 University Hospitals of Morecambe Bay, Royal Lancashire Infirmary, Lancaster, Lancashire UK; 18 Royal Berkshire NHS Foundation Trust, Reading, UK; 19 Department of Surgery, Baylor College of Medicine, Houston, TX USA; 20 Faculty of Health and Wellbeing, Department of Allied Health Professions, Sheffield Hallam University, Collegiate Cresent Campus, Sheffield, UK; 21 Division of Population Medicine, Cardiff University, Heath Park Cardiff, UK; 22 Breast Unit – Department of Medicine - The Royal Marsden NHS Foundation Trust, London, UK; 23 Breast Cancer Research Division, The Institute of Cancer Research, London, UK; 24 Department of Cardiovascular Sciences, University of Leicester, Cardiovascular Research Centre, Glenfield General Hospital, Leicester, UK; 25 EpiGenesys, University of Sheffield, Sheffield, UK; 26 University of Nottingham, Royal Derby Hospital, Derby, UK; 27 School of Psychology & Clinical Language Sciences, University of Reading, Reading, UK; 28 Cancer Epidemiology Unit, Nuffield Department of Population Health, University of Oxford, Oxford, UK; 29 Yorkshire and Humber Research Network Consumer Research Panel, Sheffield, UK; 30 Leeds General Infirmary, Leeds, West Yorkshire UK; 31 Liverpool and broadgreen Hospitals NHS Foundation Trust, Thomas Drive, Liverpool, Merseyside UK

**Keywords:** Chemotherapy, Breast cancer

## Abstract

**Background:**

Chemotherapy improves outcomes for high risk early breast cancer (EBC) patients but is infrequently offered to older individuals. This study determined if there are fit older patients with high-risk disease who may benefit from chemotherapy.

**Methods:**

A multicentre, prospective, observational study was performed to determine chemotherapy (±trastuzumab) usage and survival and quality-of-life outcomes in EBC patients aged ≥70 years. Propensity score-matching adjusted for variation in baseline age, fitness and tumour stage.

**Results:**

Three thousands four hundred sixteen women were recruited from 56 UK centres between 2013 and 2018. Two thousands eight hundred eleven (82%) had surgery. 1520/2811 (54%) had high-risk EBC and 2059/2811 (73%) were fit. Chemotherapy was given to 306/1100 (27.8%) fit patients with high-risk EBC. Unmatched comparison of chemotherapy versus no chemotherapy demonstrated reduced metastatic recurrence risk in high-risk patients(hazard ratio [HR] 0.36 [95% CI 0.19–0.68]) and in 541 age, stage and fitness-matched patients(adjusted HR 0.43 [95% CI 0.20–0.92]) but no benefit to overall survival (OS) or breast cancer-specific survival (BCSS) in either group. Chemotherapy improved survival in women with oestrogen receptor (ER)-negative cancer (OS: HR 0.20 [95% CI 0.08–0.49];BCSS: HR 0.12 [95% CI 0.03–0.44]).Transient negative quality-of-life impacts were observed.

**Conclusions:**

Chemotherapy was associated with reduced risk of metastatic recurrence, but survival benefits were only seen in patients with ER-negative cancer. Quality-of-life impacts were significant but transient.

**Trial Registration:**

ISRCTN 46099296

## Background

In 2014–2016 over 18,500 women per year aged ≥70 years were diagnosed with breast cancer in the UK, representing 34% of all diagnoses.^1^ Breast cancer survival is worse in older patients^2^ who have not experienced similar outcome improvements compared with younger individuals in the past three decades.^3^ This may reflect late presentation, more comorbidities or undertreatment. Significant treatment variations between centres are frequently reported in older adults.^4,5^ However, interpreting such data can be challenging without information on fitness, which may mitigate treatment benefits, due to competing mortality risks and increased treatment-related toxicity.

Chemotherapy benefit in older women is controversial. While there have been many high-quality randomised clinical trials (RCTs) to evaluate the impact of systemic chemotherapy, the majority of trials excluded or recruited poorly amongst older patients, and tended to enrol fitter individuals.^6^ This reflects clinicians’ and patients’ toxicity concerns and reticence from trialists about diluting the study power by introducing higher morbidity rates and competing causes of death in less fit older patients.

Older adults derive less benefit from chemotherapy compared to younger patients. Benefit is present between the ages of 70 and 80, although data for women aged over 80 years are scarce.^7^ The Bridging the Age Gap study was designed to recruit a large, real-world, cohort of older women with breast cancer including detailed baseline fitness data and information about the cancer, treatment received and outcomes. The objectives of this study analysis were to determine health status-stratified outcomes for EBC patients aged ≥70 according to whether they received guideline concordant or non-concordant care with a particular focus on chemotherapy use. In this paper, the age- and risk-stratified patterns of receipt of adjuvant systemic therapy are described in older EBC patients, with propensity score-matched analysis of disease recurrence, survival and quality-of-life outcomes.

## Methods

### Study design

Bridging the Age Gap is a prospective multicentre, observational cohort study. Patients were recruited from 56 UK centres in England and Wales (Supplementary Table 1). Eligible patients were women ≥70 years at diagnosis of primary operable invasive breast cancer (TNM stages: T1-3 (plus some T4b), N0-1, M0). Those unsuitable for surgery or with previous EBC within five years were not eligible.

### Baseline data collection

Patients were recruited at the time of EBC diagnosis and before commencing treatment and could participate at three levels: full, partial (no requirement to complete quality of life [QoL] assessments) or by proxy (simple third-party data collection for those with cognitive impairment).

Baseline data were collected about the primary tumour including; cancer type, grade, nodal status, tumour size, oestrogen (ER), progesterone (PR) and human epidermal growth factor receptor 2 (HER2) status. Staging was performed if clinically indicated. Surgical, radiotherapy and systemic therapy data were collected.

At baseline, patients underwent assessments using validated tools including: comorbidities (Charlson comorbidity index [CCI]),^8^ nutrition (Abridged Patient Generated Subjective Global Assessment [aPG-SGA]),^9,10^ functional status (Activities of Daily Living [ADL]),^11^ advanced functional status (Instrumental Activities of Daily Living [IADL]),^12^ dementia (Mini Mental State Examination [MMSE]),^13^ Eastern Cooperative Oncology Group Performance Status (ECOG PS) and medication list.

Quality-of-life was assessed using the EuroQol-5D-5L (EQ-5D-5L).^14^ Assessments on the European Organisation for the Research and Treatment of Cancer QoL Questionnaire (EORTC-QLQ)-C30,^15^ EORTC-QLQ-BR23,^16^ EORTC-QLQ-ELD15^17^ were also collected but are presented elsewhere.^18^

### Follow-up and outcomes

Patients were followed up at 6 weeks, and 6, 12, 18 and 24 months. Survival outcomes (date and cause of death) were obtained at 52 months median follow-up from the UK cancer registry. All patients were assessed for recurrence and QoL at each visit. Complications were categorised using the Common Terminology Criteria for Adverse Events system (CTCAE v4.0).

Chemotherapy-related mortality was defined as death within 30 days of chemotherapy or if chemotherapy was documented as a contributing cause. Deaths were categorised as disease related or other causes. Deaths were reviewed by the chief investigator blind to treatment decisions. Deaths were classified as disease related if the death was related to the initial breast cancer. Patients for whom the cause could not be established were excluded from cause-specific analyses.

### Statistical analyses

Analyses were performed in IBM SPSS statistics version 24 and R version 3.6.3.^19^ A *p* < 0.05 was considered statistically significant.

The relationships between systemic therapy use and tumour and patient characteristics were evaluated using uni- and multi-variable logistic regression. High-risk EBC was defined if any of the following criteria were present: node-positive, ER-negative, HER2 positive, grade 3 or Recurrence Score ≥25. (Supplementary Table 2a). Additional analyses were conducted in patients with ER-negative and HER2-positive tumours, where the benefits from chemotherapy might be anticipated. Fitness was defined based on geriatric assessments and categorised into fit, vulnerable and frail according to a cumulative score including measures of functional status, comorbidities, polypharmacy, nutritional status and cognitive status (Supplementary Table 2b).

Both overall survival (OS) and breast cancer-specific survival (BCSS) were compared in treated and untreated patients. A Cox proportional hazards model was fitted using regression-based adjustment based on covariates of: treatment; age; categories of aPG-SGA, ADL, IADL, CCI, MMSE, ECOG, medications and Nottingham Prognostic Index (NPI)^20^ and HER2 for all high-risk patients. Hazard ratios (HR) and corresponding 95% confidence intervals (CIs) were calculated.

A propensity score adjustment among sufficiently similar high-risk patients was fitted using a Cox model with a shared frailty term (or random effect) for matched patients. Participants were matched exactly on NPI category and HER2 status, and logistic regression was used to calculate propensity scores for treatment in relation to age, aPG-SGA category, ADL category, IADL category, MMSE category, CCI category, ECOG PS category and number of medications. The ratio and calliper widths of the propensity scores were chosen following examination of the propensity score overlaps for several combinations of ratios and callipers. A 1:3 ratio for chemotherapy versus no chemotherapy and a calliper of 0.25 times the propensity scores’ standard deviation was used to ensure participants were closely matched whilst retaining as many patients as possible.

The QoL questionnaires were scored according to the EQ-5D-5L User Guide (Version 3.0).^21^ Missing data were managed accordingly. The QoL analysis included only patients with high-risk EBC as detailed in Supplementary Table 2a and where questionnaires were available. The mean difference (95% CI) of the domain scores at each time-point, adjusted for baseline scores, was calculated with linear regression models for high-risk participants. Propensity score-matching was also performed, as detailed above, to compare the EQ-5D-5L usual activities score in a matched cohort receiving chemotherapy versus patients not receiving it.

## Results

**Fig. 1:**
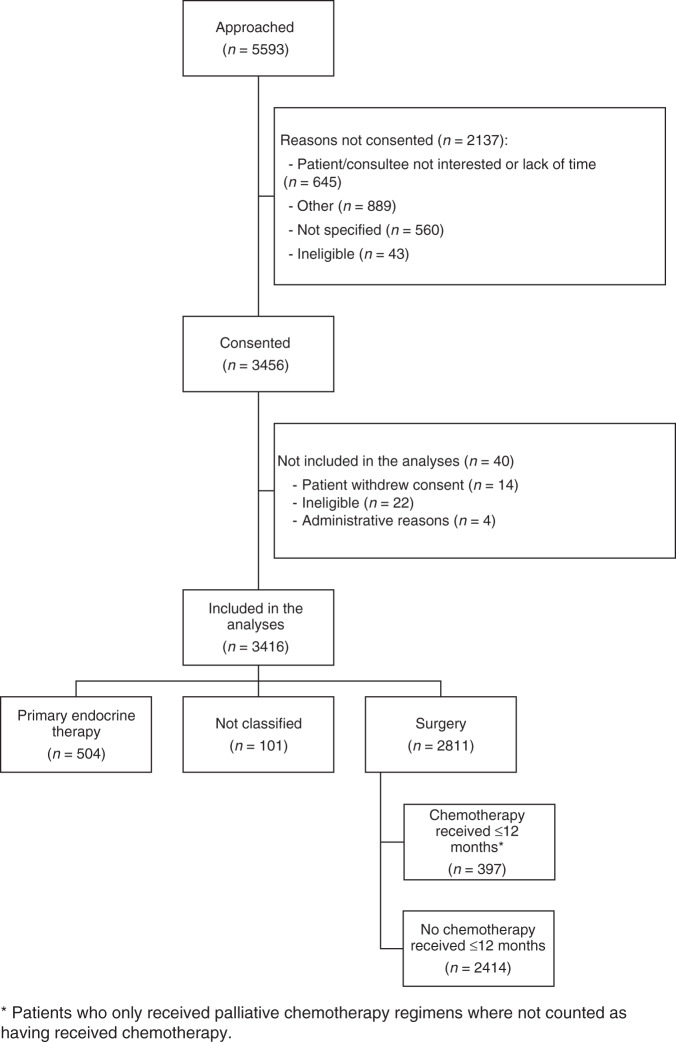
STROBE diagram. STROBE flow diagram for the chemotherapy vs no chemotherapy analyses.

Between January 2013 and June 2018, 3456 women were recruited from 56 centres in England and Wales. This analysis was restricted to the 2811 women who underwent surgery within 6 months of diagnosis (STROBE diagram [Fig. 1]).^22^ Patients’ characteristics according to geriatric assessments, tumour characteristics, postoperative histology and surgery performed are shown in Table 1.

**Table 1:** Baseline tumour, patient and treatment characteristics by age.

		70–74	75–79	80–84	≥85	All
		*N* = 1173	*N* = 899	*N* = 506	*N* = 233	*N* = 2811
Participation level	Full	926 (78.9%)	674 (75.0%)	368 (72.7%)	143 (61.4%)	2111 (75.1%)
	Partial	225 (19.2%)	209 (23.2%)	123 (24.3%)	64 (27.5%)	621 (22.1%)
	Consultee	22 (1.9%)	16 (1.8%)	15 (3.0%)	26 (11.2%)	79 (2.8%)
Main side	Right	535 (45.6%)	418 (46.5%)	247 (48.8%)	105 (45.1%)	1305 (46.4%)
	Left	638 (54.4%)	481 (53.5%)	259 (51.2%)	128 (54.9%)	1506 (53.6%)
Tumour size (mm)	≤20	649 (55.3%)	371 (41.3%)	184 (36.4%)	75 (32.2%)	1279 (45.5%)
	21–50	439 (37.4%)	439 (48.8%)	271 (53.6%)	136 (58.4%)	1285 (45.7%)
	>50	66 (5.6%)	66 (7.3%)	40 (7.9%)	16 (6.9%)	188 (6.7%)
	Unknown	19 (1.6%)	23 (2.6%)	11 (2.2%)	6 (2.6%)	59 (2.1%)
Tumour size (mm)	*n*	1154	876	495	227	2752
	Mean (SD)	23.1 (17.7)	26.5 (16.2)	27.6 (15.4)	28.8 (15.7)	25.4 (16.8)
	Median (IQR)	19.0 (12.0, 28.0)	22.0 (16.0, 32.0)	25.0 (17.0, 35.0)	25.0 (19.0, 35.0)	21.0 (15.0, 31.0)
	Min, Max	0, 210	0, 155	0, 120	7, 120	0, 210
Nodal status	pN0-1mi	867 (73.9%)	573 (63.7%)	326 (64.4%)	147 (63.1%)	1913 (68.1%)
	pN1	212 (18.1%)	223 (24.8%)	117 (23.1%)	60 (25.8%)	612 (21.8%)
	pN2	46 (3.9%)	54 (6.0%)	36 (7.1%)	11 (4.7%)	147 (5.2%)
	pN3	29 (2.5%)	25 (2.8%)	16 (3.2%)	8 (3.4%)	78 (2.8%)
	pNx	19 (1.6%)	24 (2.7%)	11 (2.2%)	7 (3.0%)	61 (2.2%)
Grade	Grade 1	199 (17.0%)	110 (12.2%)	47 (9.3%)	25 (10.7%)	381 (13.6%)
	Grade 2	635 (54.1%)	482 (53.6%)	255 (50.4%)	113 (48.5%)	1485 (52.8%)
	Grade 3	311 (26.5%)	278 (30.9%)	190 (37.5%)	86 (36.9%)	865 (30.8%)
	Unknown	28 (2.4%)	29 (3.2%)	14 (2.8%)	9 (3.9%)	80 (2.8%)
Histology	Ductal NST	761 (64.9%)	567 (63.1%)	341 (67.4%)	146 (62.7%)	1815 (64.6%)
	Lobular carcinoma	164 (14.0%)	128 (14.2%)	58 (11.5%)	25 (10.7%)	375 (13.3%)
	Tubular carcinoma	21 (1.8%)	5 (0.6%)	3 (0.6%)	0 (0.0%)	29 (1.0%)
	Mucinous carcinoma	18 (1.5%)	28 (3.1%)	12 (2.4%)	13 (5.6%)	71 (2.5%)
	Other	110 (9.4%)	83 (9.2%)	53 (10.5%)	20 (8.6%)	266 (9.5%)
	Unknown	99 (8.4%)	88 (9.8%)	39 (7.7%)	29 (12.4%)	255 (9.1%)
ER status	Negative	141 (12.0%)	117 (13.0%)	74 (14.6%)	40 (17.2%)	372 (13.2%)
	Positive	1002 (85.4%)	753 (83.8%)	414 (81.8%)	185 (79.4%)	2354 (83.7%)
	Unknown	30 (2.6%)	29 (3.2%)	18 (3.6%)	8 (3.4%)	85 (3.0%)
HER2 status	Negative	981 (83.6%)	724 (80.5%)	375 (74.1%)	192 (82.4%)	2272 (80.8%)
	Inconclusive	9 (0.8%)	7 (0.8%)	4 (0.8%)	2 (0.9%)	22 (0.8%)
	Positive	136 (11.6%)	115 (12.8%)	63 (12.5%)	18 (7.7%)	332 (11.8%)
	Unknown	47 (4.0%)	53 (5.9%)	64 (12.6%)	21 (9.0%)	185 (6.6%)
Oncotype DX test performed	No	212 (18.1%)	138 (15.4%)	76 (15.0%)	38 (16.3%)	464 (16.5%)
	**Yes**	26 (2.2%)	13 (1.4%)	2 (0.4%)	0 (0.0%)	41 (1.5%)
	Not Applicable	306 (26.1%)	265 (29.5%)	186 (36.8%)	75 (32.2%)	832 (29.6%)
	Unknown	629 (53.6%)	483 (53.7%)	242 (47.8%)	120 (51.5%)	1474 (52.4%)
Charlson comorbidity index (no age)	*n*	1133	869	481	224	2707
	Mean (SD)	0.90 (1.21)	1.10 (1.36)	1.19 (1.37)	1.09 (1.30)	1.03 (1.30)
	Median (IQR)	0.00 (0.00, 2.00)	1.00 (0.00, 2.00)	1.00 (0.00, 2.00)	1.00 (0.00, 2.00)	1.00 (0.00, 2.00)
	Min, Max	0, 6	0, 9	0, 9	0, 6	0, 9
Charlson calculated 10-year survival probability^a^	*n*	1133	869	481	224	2707
	Mean (SD)	0.55 (0.28)	0.51 (0.29)	0.28 (0.24)	0.26 (0.23)	0.47 (0.29)
	Median (IQR)	0.77 (0.21, 0.77)	0.53 (0.21, 0.77)	0.21 (0.02, 0.53)	0.21 (0.02, 0.53)	0.53 (0.21, 0.77)
	Min, Max	0, 0.77	0, 0.77	0, 0.77	0, 0.53	0, 0.77
Number of concurrent medications	*n*	973	801	462	210	2446
	Mean (SD)	3.85 (2.66)	4.16 (2.63)	4.26 (2.63)	4.21 (2.53)	4.06 (2.64)
	Median (IQR)	3.00 (2.00, 5.00)	4.00 (2.00, 6.00)	4.00 (2.00, 6.00)	4.00 (2.00, 6.00)	4.00 (2.00, 5.75)
	Min, Max	0, 14	0, 18	0, 14	0, 14	0, 18
ADL category	No dependency	924 (78.8%)	623 (69.3%)	331 (65.4%)	126 (54.1%)	2004 (71.3%)
	Mild dependency	89 (7.6%)	109 (12.1%)	67 (13.2%)	43 (18.5%)	308 (11.0%)
	Moderate/severe dependency	70 (6.0%)	101 (11.2%)	60 (11.9%)	47 (20.2%)	278 (9.9%)
	Unknown	90 (7.7%)	66 (7.3%)	48 (9.5%)	17 (7.3%)	221 (7.9%)
IADL category	No dependency	955 (81.4%)	679 (75.5%)	332 (65.6%)	103 (44.2%)	2069 (73.6%)
	Mild dependency	54 (4.6%)	78 (8.7%)	70 (13.8%)	47 (20.2%)	249 (8.9%)
	Moderate/severe dependency	67 (5.7%)	70 (7.8%)	55 (10.9%)	66 (28.3%)	258 (9.2%)
	Unknown	97 (8.3%)	72 (8.0%)	49 (9.7%)	17 (7.3%)	235 (8.4%)
MMSE category	Normal function	1059 (90.3%)	805 (89.5%)	444 (87.7%)	186 (79.8%)	2494 (88.7%)
	Mild impairment	91 (7.8%)	74 (8.2%)	50 (9.9%)	33 (14.2%)	248 (8.8%)
	Moderate impairment	11 (0.9%)	12 (1.3%)	5 (1.0%)	8 (3.4%)	36 (1.3%)
	Severe	12 (1.0%)	8 (0.9%)	7 (1.4%)	6 (2.6%)	33 (1.2%)
APG-SGA category	Low	929 (79.2%)	709 (78.9%)	370 (73.1%)	172 (73.8%)	2180 (77.6%)
	Moderate	111 (9.5%)	88 (9.8%)	62 (12.3%)	27 (11.6%)	288 (10.2%)
	High	15 (1.3%)	13 (1.4%)	10 (2.0%)	2 (0.9%)	40 (1.4%)
	Unknown	118 (10.1%)	89 (9.9%)	64 (12.6%)	32 (13.7%)	303 (10.8%)
ECOG performance status	0	930 (79.3%)	619 (68.9%)	305 (60.3%)	90 (38.6%)	1944 (69.2%)
	1	151 (12.9%)	205 (22.8%)	142 (28.1%)	109 (46.8%)	607 (21.6%)
	2	21 (1.8%)	24 (2.7%)	23 (4.5%)	12 (5.2%)	80 (2.8%)
	3	10 (0.9%)	9 (1.0%)	8 (1.6%)	9 (3.9%)	36 (1.3%)
	4	1 (0.1%)	0 (0.0%)	0 (0.0%)	0 (0.0%)	1 (0.0%)
	Unknown	60 (5.1%)	42 (4.7%)	28 (5.5%)	13 (5.6%)	143 (5.1%)
Breast surgery	Wide local excision (non wire localised)	769 (65.5%)	504 (56.1%)	236 (46.7%)	89 (38.2%)	1598 (56.8%)
	Therapeutic mammoplasty/breast reshaping after WLE	35 (3.0%)	12 (1.3%)	2 (0.4%)	2 (0.9%)	51 (1.8%)
	Mastectomy	316 (26.9%)	346 (38.5%)	251 (49.6%)	136 (58.4%)	1049 (37.3%)
	Mastectomy and reconstruction	25 (2.1%)	10 (1.1%)	2 (0.4%)	0 (0.0%)	37 (1.3%)
	Other	10 (0.9%)	5 (0.6%)	5 (1.0%)	0 (0.0%)	20 (0.7%)
	Unknown	18 (1.5%)	22 (2.4%)	10 (2.0%)	6 (2.6%)	56 (2.0%)
Axillary surgery	Axillary sample	38 (3.2%)	30 (3.3%)	11 (2.2%)	9 (3.9%)	88 (3.1%)
	Axillary clearance	134 (11.4%)	134 (14.9%)	99 (19.6%)	47 (20.2%)	414 (14.7%)
	Sentinel lymph node biopsy	881 (75.1%)	633 (70.4%)	336 (66.4%)	130 (55.8%)	1980 (70.4%)
	Internal mammary node biopsy	0 (0.0%)	1 (0.1%)	0 (0.0%)	0 (0.0%)	1 (0.0%)
	No axillary surgery	23 (2.0%)	16 (1.8%)	22 (4.3%)	19 (8.2%)	80 (2.8%)
	Unknown	97 (8.3%)	85 (9.5%)	38 (7.5%)	28 (12.0%)	248 (8.8%)

^a^Ten-year survival calculated as 0.983^(e^CCI × 0.9^), where *CCI* Charlson Comorbidity Index.

Of the 2811 patients, 397 (14.1%) received chemotherapy (365 [92%] in the adjuvant setting, 30 [8%] in neoadjuvant setting, and 2 [0.5%] unknown). Of those 380 patients for whom the chemotherapy regimen received was known, 132 (34.7%) received an anthracycline-taxane combination, 124 (32.6%) a taxane (without anthracycline), 123 (32.4%) an anthracycline and 1 CMF. 332 patients (11.8%) had HER2-positive EBC. Of these patients, 150 (45.1%) received chemotherapy plus trastuzumab, 13 (3.9%) trastuzumab without chemotherapy, and 9 (2.7%) chemotherapy without trastuzumab. Overall, 1753/2811 (62.4%) patients received radiotherapy and 2239/2354 (95.1%) ER-positive patients received endocrine therapy.

Chemotherapy receipt according to tumour and patient characteristics is shown in Supplementary Tables 3 and 4. Univariate and multivariate analyses are shown in Table 2. Younger, less dependent patients with high-risk tumours and with fewer comorbidities were more likely to receive chemotherapy.

**Table 2:** Relationship between chemotherapy use and patient characteristics: univariate (Table 2a) and multi-variable (Table 2b) analyses.

(a) Results for univariate logistic regression models.			
Variable	Level	OR (95% CI)	*P*-value
Age		0.84 (0.82, 0.87)	<0.001
ADL score		1.07 (1.04, 1.11)	<0.001
IADL score		1.77 (1.43, 2.25)	<0.001
CCI (no age)		0.84 (0.77, 0.93)	<0.001
APG-SGA		0.95 (0.89, 1.01)	0.127
Allred score		0.80 (0.78, 0.83)	<0.001
Tumour grade	Grade 1	–	–
	Grade 2	9.04 (3.78, 29.58)	<0.001
	Grade 3	37.67 (15.87, 122.76)	<0.001
ER positive		0.22 (0.17, 0.28)	<0.001
HER2 status^a^	Negative	–	–
	Positive	8.49 (6.57, 10.97)	<0.001
MMSE category	Normal function	–	–
	Mild impairment	0.75 (0.49, 1.11)	0.172
	Moderate impairment	1.18 (0.44, 2.67)	0.711
	Severe	0.38 (0.06, 1.27)	0.188
Nodal status^b^	pN0-1mi	–	–
	pN1	2.18 (1.69, 2.80)	<0.001
	pN2	5.05 (3.47, 7.29)	<0.001
	pN3	6.42 (3.96, 10.30)	<0.001

(b) Results from the multi-variable logistic regression model.			
Variable	Level	OR (95% CI)	*P*-value
Age		0.74 (0.71, 0.78)	<0.001
IADL score		1.97 (1.53, 2.63)	<0.001
CCI (no age)		0.83 (0.73, 0.95)	0.007
Tumour grade	Grade 1	–	–
	Grade 2	8.42 (3.05, 34.90)	<0.001
	Grade 3	29.50 (10.59, 123.00)	<0.001
ER positive		0.19 (0.13, 0.28)	<0.001
HER2 status	Negative	–	–
	Positive	8.94 (6.19, 13.01)	<0.001
Nodal status	pN0-1mi		
	pN1	4.01 (2.81, 5.75)	<0.001
	pN2	11.24 (6.43, 19.74)	<0.001
	pN3	8.84 (4.31, 18.05)	<0.001

^a^Tests marked as ‘Inconclusive’ were removed from this analysis.

^b^Those with nodal status pNx were removed from this analysis.

High-risk tumours were present in 1520 (54%) patients and 376/1520 (25%) received chemotherapy compared with 21/1291 (1.6%) of patients with non-high-risk tumours (Table 3a). 2059 patients (73%) were fit and 752 vulnerable or frail (27%) (Table 3b). Of those who were fit, 1100 also had high-risk EBC, and of these patients 306 (28%) received chemotherapy (Table 3c).

**Table 3:** Chemotherapy use according to risk of recurrence and fitness.

(a) Use of chemotherapy by risk status.			
Risk	Chemotherapy	No Chemotherapy	Total
High risk	376 (24.7%)	1144 (75.3%)	**1520 (100.0%)**
Non-high risk	21 (1.6%)	1270 (98.4%)	**1291 (100.0%)**
Total	**397 (14.1%)**	**2414 (85.9%)**	**2811 (100.0%)**

(b) Use of chemotherapy by fitness.			
Fitness	Chemotherapy	No Chemotherapy	Total
Fit	322 (15.6%)	1737 (84.4%)	**2059 (100.0%)**
Vulnerable	75 (10.0%)	675 (90.0%)	**750 (100.0%)**
Frail	0 (0.0%)	2 (100.0%)	**2 (100.0%)**
Total	**397 (14.1%)**	**2414 (85.9%)**	**2811 (100.0%)**

(c) Use of chemotherapy by risk and fitness.					
Fitness	High risk		Non-high risk		Total
	Chemotherapy	No chemotherapy	Chemotherapy	No chemotherapy	
Fit	306 (14.9%)	794 (38.6%)	16 (0.8%)	943 (45.8%)	**2059 (100.0%)**
Vulnerable	70 (9.3%)	349 (46.5%)	5 (0.7%)	326 (43.5%)	**750 (100.0%)**
Frail	0 (0.0%)	1 (50%)	0 (0.0%)	1 (50%)	**2 (100%)**
Total	**376 (13.4%)**	**1144 (40.7%)**	**21 (0.7%)**	**1270 (45.2%)**	**2811 (100.0%)**

Bold values represent the total numbers for each column or row.

**Fig. 2:**
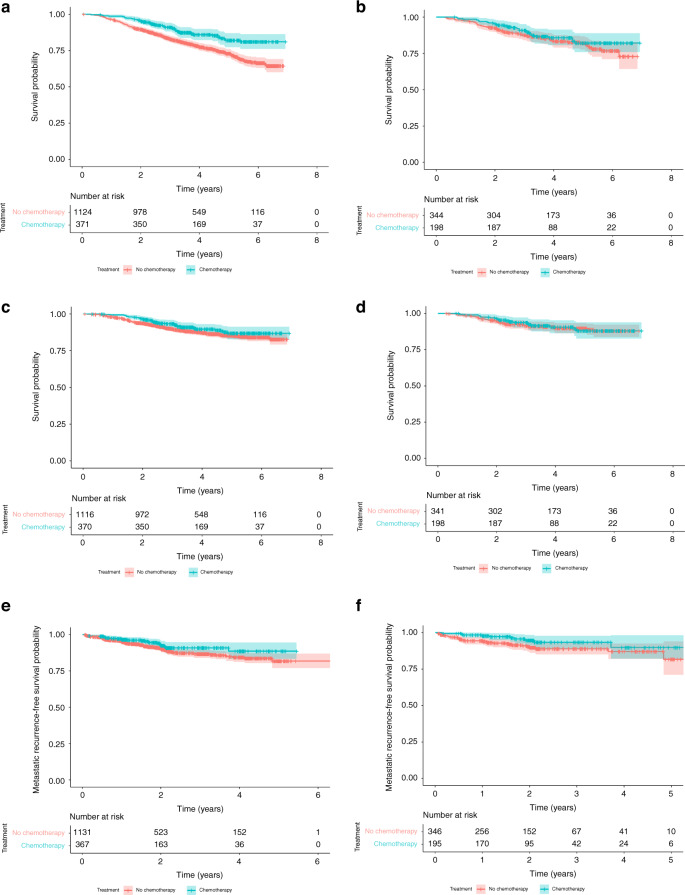
Kaplan–Meier plots of survival and metastatic recurrence outcomes. **a** Overall Survival in unmatched high-risk patients (*n* = 1495). Adjusted HR 0.87 (95% CI 0.58–1.28, *p* = 0.47). **b** Overall survival in matched high-risk patients (*n* = 542). Adjusted HR 0.79 (95% CI: 0.50–1.26, *p* = 0.32). **c** Breast cancer-specific survival in unmatched high-risk patients (*n* = 1486). Adjusted HR 0.92 (95% CI: 0.56–1.53, *p* = 0.76). **d** Breast cancer-specific survival in matched high-risk patients (*n* = 539). Adjusted HR 0.93 (95% CI: 0.52–1.66, *p* = 0.80). **e** Metastatic recurrence in unmatched high-risk patients (*n* = 1498). Adjusted HR 0.36 (95% CI: 0.19–0.68, *p* = 0.002). **f** Metastatic recurrence in matched high-risk patients (*n* = 541). Adjusted HR 0.53 (95% CI: 0.26–1.07, *p* = 0.08).

At a median follow-up of 52 months, mortality status was available for 98% (1495/1520) of high-risk patients (371 in the chemotherapy group, 1124 in the no chemotherapy group). Chemotherapy was associated with a longer OS, but the difference was not statistically significant when adjusted for other covariates (unadjusted HR 0.55 [95% CI 0.40–0.73, *p* < 0.001] and adjusted HR 0.87 [95% CI 0.58–1.28, *p* = 0.469] (Fig. 2a). In a propensity score-matched analysis 200 patients receiving chemotherapy were matched to 350 who did not receive it. Supplementary Table 5 shows the characteristics of the matched dataset and the matching process and quality are summarised in Supplementary Fig. 1. Mortality status was available for 542 (99%) of the matched patients. Chemotherapy was associated with a longer OS although this was not statistically significant (HR 0.79 [95% CI 0.50–1.26, *p* = 0.320]) (Fig. 2b).

BCSS was available for 98% (1486/1520) of patients in the high-risk population. Chemotherapy was not associated with improved BCSS (unadjusted HR 0.76 [95% CI 0.53–1.10, *p* = 0.147] and adjusted HR 0.92 [95% CI 0.56–1.53, *p* = 0.758]) (Fig. 2c). In the propensity score-matched population, BCSS was available for 539 patients (98%). Chemotherapy was also not found to be associated with improved BCSS (HR 0.93 [95% CI 0.52–1.66, *p* = 0.798]) (Fig. 2d).

Metastatic recurrence data were available for 1498 high-risk patients (99%). Chemotherapy was associated with a significantly lower risk of metastatic recurrence in the unmatched population (unadjusted HR 0.67 [95% CI 0.43–1.04, *p* = 0.077] and adjusted HR 0.36 [95% CI 0.19–0.68, *p* = 0.002]) (Fig. 2e). In 541 matched patients (98%), chemotherapy was also associated with a lower metastatic recurrence risk (HR 0.53 [95% CI 0.26–1.07, *p* = 0.076]) (Fig. 2f).

**Fig. 3:**
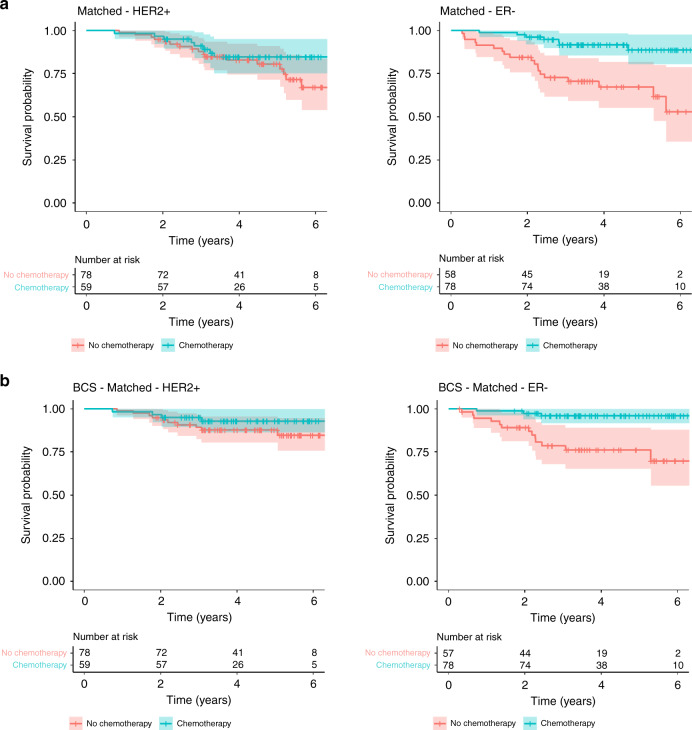
Kaplan–Meier plots for survival outcomes in matched patients with HER2-positive or ER-negative breast cancer. **a** Overall survival in patients with HER2-positive breast cancer (*n* = 137): HR 0.63 [0.27–1.48]; and in patients with ER-negative breast cancer (*n* = 136): HR 0.20 [0.08–0.49]. **b** Breast cancer-specific survival in patients with HER2-positive breast cancer (*n* = 137): HR 0.5 [0.16–1.63]; and in patients with ER-negative breast cancer (*n* = 135): HR 0.12 [0.03–0.44].

Additional post-hoc exploratory analyses were performed in disease subgroups. Out of 369 patients with ER-negative EBC and known mortality status, 132 (35.8%) received chemotherapy. In a propensity score-matched analysis in 136 patients, chemotherapy was associated with better OS (HR 0.20 [0.08–0.49]) and BCSS (HR 0.12 [0.03–0.44]) (Fig. 3, Supplementary Table 6 and Supplementary Fig. 2). Three hundred twenty six patients with HER2-positive EBC and known mortality status of whom 156 (47.9%) received chemotherapy with or without trastuzumab. Fewer deaths from breast cancer and other causes occurred in those receiving chemotherapy with or without trastuzumab. However, in a matched analysis in 137 patients, the differences were not statistically significant for OS (HR 0.63 [0.27–1.48]) or BCSS (HR 0.50 ([0.16–1.63]) (Fig. 3, Supplementary Table 6 and Supplementary Fig. 2).

Supplementary Table 7 outlines chemotherapy toxicity. Among 397 patients receiving chemotherapy, there was one chemotherapy-related death (0.25%) (due to congestive heart failure) and 132 (33.2%) had an episode of infection, which was grade 3 or 4 in 50 (12.6%). Among the 163 patients who received trastuzumab, 4 (2.5%) experienced cardiac failure within the first 6 months and 12 (6.7%) within the first year.

Among 2811 patients undergoing surgery, the QoL analysis was restricted to 1520/2811 (54.1%) with high-risk EBC of whom 1315/1520 (86.5%) had an EQ-5D-5L score available at baseline. Of these patients, 376/1520 (24.7%) received chemotherapy. Health utilities were similar with estimated mean differences less than 0.02 units (*p* > 0.1), whereas the visual analogue scale (VAS) measures were significantly worse at 6 months in patients receiving chemotherapy versus not (adjusted mean difference −6.57, 95% CI −8.74 to −4.40, *p* < 0.001). Changes were no longer significant at 12 months and thereafter (Supplementary Table 8; Supplementary Fig. 3).

A similar pattern on EQ-5D-5L usual activities score was seen in 520 propensity score-matched patients (including 118 patients receiving chemotherapy and 332 not receiving it) (Supplementary Fig. 4).

## Discussion

This study represents one of the largest prospective cohort studies conducted in older women with breast cancer and provides valuable data on tumour characteristics and health of older EBC patients. As expected, the majority of patients had relatively good prognosis tumours, with relatively low rates of nodal involvement and adverse biology as determined by ER and HER2 status. Nonetheless, there remained a substantial proportion of high risk, fit patients (on baseline assessments), with a high relapse risk in their expected lifetime. Ensuring that these patients receive adequate treatment is a priority for clinicians.

A key finding of this study is that 27.8% of fit high-risk EBC older patients received chemotherapy. In the ACheW study 30% of high-risk EBC patients were offered chemotherapy and 17% received it.^23^ Analyses of European and US registry data report similar findings.^5,24,25^ These analyses did not consider recurrence risk (as determined by histopathological variables) and patients’ fitness (to not only receive treatment but also to live long enough to benefit). The current study overcame these limitations, by defining recurrence risk and fitness, and still demonstrates low chemotherapy uptake. This may be due to uncertainty on chemotherapy benefit in older adults, toxicity concerns and patients’ and carers’ choice.

In order to investigate the survival benefits of chemotherapy for older EBC patients, we conducted survival analyses in those at high risk of recurrence. Ideally this question should be addressed by RCTs. Recruiting older patients into RCTs comparing different chemotherapy regimens is feasible,^26^ but trials comparing chemotherapy with no chemotherapy have failed to recruit.^27,28^ Moreover, older patients enrolled in RCTs may be fitter and not necessarily representative of a real-world population.^6^ In contrast, this cohort study recruited well, and recruited patients with a broad fitness range.

Our analyses attempted to correct for confounders, specifically the fact that younger, fitter patients might be more likely to receive chemotherapy, but also are biologically more likely to survive longer irrespective of chemotherapy effect. This effect is perhaps most apparent when comparing the unmatched and matched OS analyses (Fig. 2a, b).

In the high-risk population chemotherapy reduced the risks of metastatic recurrence, which did not translate into better survival. This may be because the benefit was modest and the fact that median OS for ER-positive metastatic disease patients often exceeds 3 years with contemporary therapies.^29^ Irrespective, a reduction in metastatic relapses, with their symptomatic, psychological and financial implications, may be sufficient grounds on which to offer treatment even in the absence of a survival benefit. Longer term follow-up will be required to further explore this.

Chemotherapy benefits are small for most ER-positive, HER2-negative EBC patients. Therefore, we performed exploratory analyses in patients with the more chemotherapy-sensitive subtypes, i.e. ER-negative and HER2-positive disease. In ER-negative EBC patients there was an apparent reduction of breast cancer deaths with chemotherapy. These data are consistent with an US SEER analysis suggesting that adjuvant chemotherapy benefit in older patients were restricted to those with ER-negative disease.^28,30^ In HER2-positive EBC patients, fewer breast cancer deaths occurred in those who received chemotherapy with or without trastuzumab although the differences were not statistically significant in a matched analysis. This could be explained by the small numbers in this subgroup analysis. However, a retrospective study demonstrated that HER2-positive EBC older patients do not have inferior long-term outcomes compared with younger adults not receiving chemotherapy.^31^ Low Ki67 and high bcl2 expression in the older cohort of HER2-positive patients might explain this better prognosis and also relative chemo-resistance.^31^

Our study found that mortality rates from chemotherapy were very low and side effects consistent with previous analyses.^32^ Follow-up of the cohort is planned at 10 years and may provide data about longer term benefits, although it should be recognised that with longer follow-up competing mortality causes are likely have a greater impact.

Our analysis also demonstrates that chemotherapy has a significant negative impact at 6 months on QoL, which is a meaningful endpoint in the context of a more limited survival benefit and increased risk of toxicities in this population. However, this effect resolves at 12 months consistent with previous findings in smaller or younger cohorts of patients^33,34^ and is described in a more extensive analysis performed on this patient cohort.^18^

A key strength of this study is that patients were recruited from a broad range of academic and general centres across the UK, and were likely to reflect contemporary practice and outcomes. However, despite the inclusive entry criteria and low level of intervention there was still the possibility of selection bias. In a separate analysis of this study we found that patients who did not enter the trial following screening were older and had worse functional ability.^35^ Also, as patients were not randomised, unmeasured variables might have influenced our findings despite propensity score matching. The extent to which these data reflect practice and outcomes outside of the UK is unknown, although some published data do appear comparable.^24,25^

In summary, this study demonstrates that there are a significant number of older but fit patients with high-risk EBC who are not receiving adjuvant chemotherapy. Some of these patients, particularly those with ER-negative disease, may derive benefit from chemotherapy. Clearly the benefits need to be discussed in the context of potential side effects and the transient negative impact on QoL. Nonetheless, it is important that individualised treatment decisions and discussions are made to ensure the best outcomes for older adults.

## Supplementary information

Supplemental materials

## Data Availability

The datasets generated during and/or analysed during the current study are available from the corresponding author on reasonable request.
