# Supplementary material for: Bridging The Age Gap: observational cohort study of effects of chemotherapy and trastuzumab on recurrence, survival and quality of life in older women with early breast cancer
Source: Br J Cancer. 2021 May 10;125(2):209–19. doi: 10.1038/s41416-021-01388-9 (PMC8292504; doi:10.1038/s41416-021-01388-9)

**Supplementary materials**

**Supplementary Table 1 - List of participating sites.**

|  | Site Name | Local PI |
| --- | --- | --- |
| 1 | Sheffield | Lynda Wyld and Matt Winter |
| 2 | Barnsley | Julia Dicks |
| 3 | Doncaster | Clare Rogers |
| 4 | Milton Keynes | Amanda Taylor |
| 5 | Scunthorpe and Grimsby | Rajesh Vijh (Scunthorpe), Jenny Smith (Grimsby) |
| 6 | Leicester | Monika Kaushik |
| 7 | Derby | Kwok Leung Cheung |
| 8 | East Lancashire | Julie Iddon |
| 9 | Harrogate | Matthew Adelekan |
| 10 | St Helens and Knowsley | Riccardo Audisio |
| 11 | York | Rana Nasr (York and Scarborough) |
| 12 | Liverpool | Chris Holcombe |
| 13 | Airedale | Claire Murphy |
| 14 | Leeds | Kieran Horgan |
| 15 | Bradford | Rick Linforth |
| 16 | Cardiff | Helen Sweetland |
| 17 | Aneurin Bevan Health Board | Simon Waters (Royal Gwent), Theresa Howe (Nevill Hall) |
| 18 | Royal Lancaster | Rishi Parmeshwar |
| 19 | Coventry | Abigail Tomlins |
| 20 | Grantham | Anzors Gvaramadze |
| 21 | Lincoln | Anzors Gvaramadze |
| 22 | Pilgrim | Anzors Gvaramadze |
| 23 | Hull | Peter Kneeshaw |
| 24 | Nottingham | Lisa Whisker |
| 25 | Southport | Anwar Haq |
| 26 | Leighton | Vanessa Pope |
| 27 | Royal Marsden | Jenny Rusby |
| 28 | Cheltenham General | Sarah Vestey |
| 29 | Guys and St Thomas | Michael Douek |
| 30 | Dorset County | Caroline Osborne |
| 31 | Mid Essex | Sascha Miles-Dua |
| 32 | Mid Yorkshire | Jay Naik |
| 33 | Bristol | Zoe Winters |
| 34 | Chesterfield | Iman Azmy |
| 35 | Rotherham | Inder Kumar |
| 36 | Darent Valley | Seema Seetharam |
| 37 | Kingston | Karyn Shenton |
| 38 | Colchester | Mukesh Mukesh |
| 39 | Yeovil | Caroline Osborne |
| 40 | Croydon | Sanjay Joshi |
| 41 | North Tees | Colm Hennessy |
| 42 | South Tees | Imtiaz Cheema |
| 43 | Luton and Dunstable | Mei-Lin Ah-See |
| 44 | Weston General | Rachel Ainsworth |
| 45 | Tameside | Stephanie Ridgway |
| 46 | Macclesfield | Lisa Barraclough |
| 47 | Wrightington, Wigan and Leigh | Angela Power |
| 48 | Birmingham | Fiona Hoar |
| 49 | Kings Mill | Rebecca Boulton |
| 50 | Wythenshawe | Nigel Bundred |
| 51 | Aintree | Peter Robson |
| 52 | Brighton | Gargi Patel |
| 53 | St Margaret’s | Ashraf Patel |
| 54 | St Marys | Steve Parker |
| 55 | Oxford | Asha Adwani |
| 56 | Frimley and Wexham | Ruth Davis (Wexham), Raouf Daoud (Frimley) |

**Supplementary Table 2a - Definition of risk of breast cancer recurrence based on tumour characteristics on diagnostic biopsy or surgical specimen.**

| Tumour characteristics | Risk of recurrence | |
| --- | --- | --- |
|  | **High*** | **Low** |
| ER | Negative | Positive |
| HER2 | Positive | Negative |
| Grade | 3 | 1-2 |
| Nodal involvement | Yes | No |
| Oncotype DX Recurrence Score | ≥25 | <25 |

**high risk is defined by the presence of ≥1 of the features enlisted.*

**Supplementary Table 2b – Definition of fitness categories were determined. Overall scores were defined: Fit: 0-2. Vulnerable: 3-8. Frail: 9+.**

| Domain | Range | Score | | |
| --- | --- | --- | --- | --- |
|  |  | **0** | **1** | **2** |
| ECOG PS | **0-4** | 0-1 | 2 | 3-4 |
| ADL | **0-10** | 20 | 19 | ≤18 |
| IADL | **0-8** | 8 | 7 | ≤6 |
| Charlson comorbidity index | **-** | 0-1 | - | ≥2 |
| Prescribed medications (excluding vitamins/minerals) | **-** | ≤3 | ≥4 | - |
| APSGA |  | 0-3 | 4-8 | ≥9 |
| MMSE | **0-30** | ≥24 | 20-24 | <20 |

**Supplementary Table 3 - Baseline patient characteristics by receipt of chemotherapy.**

|  | | No Chemotherapy | Chemotherapy |
| --- | --- | --- | --- |
|  |  | N = 2414 | N = 397 |
| **Participation level** | **Full** | 1789 (74.1%) | 322 (81.1%) |
|  | **Partial** | 550 (22.8%) | 71 (17.9%) |
|  | **Consultee** | 75 (3.1%) | 4 (1.0%) |
| **Age** | **n** | 2414 | 397 |
|  | **Mean (SD)** | 76.98 (5.25) | 73.62 (3.30) |
|  | **Median (IQR)** | 76.00 (73.00, 80.00) | 73.00 (71.00, 76.00) |
|  | **Min, Max** | 69, 95 | 69, 87 |
| **Charlson comorbidity index (no age)** | **n** | 2322 | 385 |
|  | **Mean (SD)** | 1.07 (1.33) | 0.81 (1.10) |
|  | **Median (IQR)** | 1.00 (0.00, 2.00) | 0.00 (0.00, 2.00) |
|  | **Min, Max** | 0, 9 | 0, 6 |
| **Charlson calculated probability** | **n** | 2322 | 385 |
|  | **Mean (SD)** | 0.45 (0.30) | 0.56 (0.27) |
|  | **Median (IQR)** | 0.53 (0.21, 0.77) | 0.77 (0.21, 0.77) |
|  | **Min, Max** | 0, 0.77 | 0, 0.77 |
| **Number of concurrent medications** | **n** | 2116 | 330 |
|  | **Mean (SD)** | 4.13 (2.66) | 3.63 (2.49) |
|  | **Median (IQR)** | 4.00 (2.00, 6.00) | 3.00 (2.00, 5.00) |
|  | **Min, Max** | 0, 18 | 0, 14 |
| **ADL category** | **No dependency** | 1683 (69.7%) | 321 (80.9%) |
|  | **Mild dependency** | 274 (11.4%) | 34 (8.6%) |
|  | **Moderate/severe dependency** | 262 (10.9%) | 16 (4.0%) |
|  | **Unknown** | 195 (8.1%) | 26 (6.5%) |
| **IADL category** | **No dependency** | 1737 (72.0%) | 332 (83.6%) |
|  | **Mild dependency** | 221 (9.2%) | 28 (7.1%) |
|  | **Moderate/severe dependency** | 248 (10.3%) | 10 (2.5%) |
|  | **Unknown** | 208 (8.6%) | 27 (6.8%) |
| **MMSE category** | **Normal function** | 2133 (88.4%) | 361 (90.9%) |
|  | **Mild impairment** | 220 (9.1%) | 28 (7.1%) |
|  | **Moderate impairment** | 30 (1.2%) | 6 (1.5%) |
|  | **Severe** | 31 (1.3%) | 2 (0.5%) |
| **APG SGA category** | **Low** | 1864 (77.2%) | 316 (79.6%) |
|  | **Moderate** | 249 (10.3%) | 39 (9.8%) |
|  | **High** | 36 (1.5%) | 4 (1.0%) |
|  | **Unknown** | 265 (11.0%) | 38 (9.6%) |
| **ECOG performance status** | **0** | 1632 (67.6%) | 312 (78.6%) |
|  | **1** | 544 (22.5%) | 63 (15.9%) |
|  | **2** | 77 (3.2%) | 3 (0.8%) |
|  | **3** | 34 (1.4%) | 2 (0.5%) |
|  | **4** | 1 (0.0%) | 0 (0.0%) |
|  | **Unknown** | 126 (5.2%) | 17 (4.3%) |

**Supplementary Table 4 - Postoperative tumour characteristics by receipt of chemotherapy.**

|  | | No Chemotherapy | Chemotherapy |
| --- | --- | --- | --- |
|  |  | N = 2414 | N = 397 |
| **Main side** | **Right** | 1128 (46.7%) | 177 (44.6%) |
|  | **Left** | 1286 (53.3%) | 220 (55.4%) |
| **Tumour size (mm)** | **n** | 2365 | 387 |
|  | **Mean (SD)** | 24.2 (15.8) | 32.8 (20.5) |
|  | **Median (IQR)** | 20.0 (14.0, 30.0) | 29.0 (21.0, 40.0) |
|  | **Min, Max** | 0, 155 | 0, 210 |
| **Tumour size (mm)** | **≤ 20** | 1183 (49.0%) | 96 (24.2%) |
|  | **21-50** | 1043 (43.2%) | 242 (61.0%) |
|  | **> 50** | 139 (5.8%) | 49 (12.3%) |
|  | **Unknown** | 49 (2.0%) | 10 (2.5%) |
| **Nodal status** | **pN0-1mi** | 1726 (71.5%) | 187 (47.1%) |
|  | **pN1** | 495 (20.5%) | 117 (29.5%) |
|  | **pN2** | 95 (3.9%) | 52 (13.1%) |
|  | **pN3** | 46 (1.9%) | 32 (8.1%) |
|  | **pNx** | 52 (2.2%) | 9 (2.3%) |
| **Grade** | **Grade 1** | 377 (15.6%) | 4 (1.0%) |
|  | **Grade 2** | 1355 (56.1%) | 130 (32.7%) |
|  | **Grade 3** | 618 (25.6%) | 247 (62.2%) |
|  | **Unknown** | 64 (2.7%) | 16 (4.0%) |
| **Histology** | **Ductal NST** | 1534 (63.5%) | 281 (70.8%) |
|  | **Lobular carcinoma** | 321 (13.3%) | 54 (13.6%) |
|  | **Tubular carcinoma** | 29 (1.2%) | 0 (0.0%) |
|  | **Mucinous carcinoma** | 70 (2.9%) | 1 (0.3%) |
|  | **Other** | 235 (9.7%) | 31 (7.8%) |
|  | **Unknown** | 225 (9.3%) | 30 (7.6%) |
| **ER positive** | **Negative** | 240 (9.9%) | 132 (33.2%) |
|  | **Positive** | 2101 (87.0%) | 253 (63.7%) |
|  | **Unknown** | 73 (3.0%) | 12 (3.0%) |
| **HER2 status** | **Negative** | 2050 (84.9%) | 222 (55.9%) |
|  | **Inconclusive** | 19 (0.8%) | 3 (0.8%) |
|  | **Positive** | 173 (7.2%) | 159 (40.1%) |
|  | **Unknown** | 172 (7.1%) | 13 (3.3%) |
| **Oncotype Dx test performed** | **No** | 428 (17.7%) | 36 (9.1%) |
|  | **Yes** | 35 (1.4%) | 6 (1.5%) |
|  | **Not Applicable** | 571 (23.7%) | 261 (65.7%) |
|  | **Unknown** | 1380 (57.2%) | 94 (23.7%) |
| **Breast surgery** | **Wide local excision** | 1433 (59.4%) | 165 (41.5%) |
|  | **Therapeutic mammoplasty / breast reshaping after WLE** | 33 (1.4%) | 18 (4.5%) |
|  | **Mastectomy** | 860 (35.6%) | 189 (47.6%) |
|  | **Mastectomy and reconstruction** | 25 (1.0%) | 12 (3.0%) |
|  | **Other** | 16 (0.7%) | 4 (1.0%) |
|  | **Unknown** | 47 (1.9%) | 9 (2.3%) |
| **Axillary surgery** | **Axillary sample** | 76 (3.1%) | 12 (3.0%) |
|  | **Axillary clearance** | 274 (11.4%) | 140 (35.3%) |
|  | **Sentinel lymph node biopsy** | 1770 (73.3%) | 210 (52.9%) |
|  | **Internal mammary node biopsy** | 1 (0.0%) | 0 (0.0%) |
|  | **No axillary surgery** | 73 (3.0%) | 7 (1.8%) |
|  | **Unknown** | 220 (9.1%) | 28 (7.1%) |

**Supplementary Table 5 -** **Covariate balance in the final matched dataset: chemotherapy vs no chemotherapy.**

|  | | Chemotherapy | No Chemotherapy |
| --- | --- | --- | --- |
|  |  | N = 200 | N = 350 |
| **Age** | **n** | 200 | 350 |
|  | **Mean (SD)** | 73.48 (2.91) | 74.36 (3.06) |
|  | **Median (IQR)** | 73.00 (71.00, 76.00) | 74.00 (72.00, 77.00) |
|  | **Min, Max** | 70, 80 | 69, 80 |
| **aPG-SGA** | **Low** | 173 (86.5%) | 302 (86.3%) |
|  | **Moderate** | 24 (12.0%) | 45 (12.9%) |
|  | **High** | 3 (1.5%) | 3 (0.9%) |
| **ADL** | **No dependency** | 162 (81.0%) | 273 (78.0%) |
|  | **Mild dependency** | 26 (13.0%) | 46 (13.1%) |
|  | **Moderate/severe dependency** | 12 (6.0%) | 31 (8.9%) |
| **iADL** | **No dependency** | 174 (87.0%) | 299 (85.4%) |
|  | **Mild dependency** | 19 (9.5%) | 31 (8.9%) |
|  | **Moderate/severe dependency** | 7 (3.5%) | 20 (5.7%) |
| **MMSE** | **Normal function** | 182 (91.0%) | 317 (90.6%) |
|  | **Mild impairment** | 15 (7.5%) | 28 (8.0%) |
|  | **Moderate impairment** | 3 (1.5%) | 5 (1.4%) |
| **CCI** | **0-1** | 176 (88.0%) | 301 (86.0%) |
|  | **> 2** | 24 (12.0%) | 49 (14.0%) |
| **Medications** | **3 or fewer** | 109 (54.5%) | 172 (49.1%) |
|  | **4 or more** | 91 (45.5%) | 178 (50.9%) |
| **ECOG** | **Low** | 195 (97.5%) | 339 (96.9%) |
|  | **Moderate** | 3 (1.5%) | 6 (1.7%) |
|  | **High** | 2 (1.0%) | 5 (1.4%) |
| **NPI** | **Moderate** | 137 (68.5%) | 245 (70.0%) |
|  | **Good** | 10 (5.0%) | 17 (4.9%) |
|  | **Poor** | 53 (26.5%) | 88 (25.1%) |
| **HER2** | **Negative** | 139 (69.5%) | 269 (76.9%) |
|  | **Positive** | 61 (30.5%) | 81 (23.1%) |

**Supplementary Table 6 - Mortality status for HER2-positive patients and ER-negative patients by use of chemotherapy.**

|  |  | No Chemotherapy | Chemotherapy | Total |
| --- | --- | --- | --- | --- |
| **HER2+** | **n** | 170 | 156 | 326 |
|  | **Died** | 45 (26.5%) | 19 (12.2%) | 64 (19.6%) |
|  | **n** | 169 | 156 | 325 |
|  | **Died of breast cancer** | 24 (14.2%) | 12 (7.7%) | 36 (11.1%) |
| **ER-** | **n** | 237 | 132 | 369 |
|  | **Died** | 92 (38.8%) | 20 (15.2%) | 112 (30.4%) |
|  | **n** | 234 | 131 | 365 |
|  | **Died of breast cancer** | 56 (23.9%) | 13 (9.9%) | 69 (18.9%) |

**Supplementary Table 7 - Toxicity in patients receiving chemotherapy (n=397)**

**Supplementary Table 7a – Adverse event rates according in the overall chemotherapy population and according to level of participation.**

|  | Consultee | Full | Partial | Total |
| --- | --- | --- | --- | --- |
|  | N = 4 | N = 322 | N = 71 | N = 397 |
| Allergic reaction to chemotherapy agents | 0 (0%) | 21 (7%) | 5 (7%) | 26 (7%) |
| Anaemia | 2 (50%) | 69 (21%) | 14 (20%) | 85 (21%) |
| Fatigue | 3 (75%) | 231 (72%) | 49 (69%) | 283 (71%) |
| Hair thinning | 2 (50%) | 205 (64%) | 42 (59%) | 249 (63%) |
| Infection | 1 (25%) | 103 (32%) | 28 (39%) | 132 (33%) |
| Low white cell count | 2 (50%) | 76 (24%) | 19 (27%) | 97 (24%) |
| Nausea | 3 (75%) | 134 (42%) | 30 (42%) | 167 (42%) |
| Thrombocytopenia | 3 (75%) | 19 (6%) | 7 (10%) | 29 (7%) |

**Supplementary Table 7b - Adverse event rates according to CTCAE grading.**

|  | Worse CTCAE grading | Individuals |
| --- | --- | --- |
| Allergic reactions | 1 | 7/26 (26.9%) |
|  | 2 | 9/26 (34.6%) |
|  | 3 | 2/26 (7.7%) |
|  | 4 | 1/26 (3.8%) |
|  | Missing | 7/26 (26.9%) |
| Anaemia | 1 | 29/85 (34.1%) |
|  | 2 | 25/85 (29.4%) |
|  | 3 | 2/85 (2.4%) |
|  | 4 | 1/85 (1.2%) |
|  | Missing | 28/85 (32.9%) |
| Fatigue | 1 | 95/283 (33.6%) |
|  | 2 | 75/283 (26.5%) |
|  | 3 | 26/283 (9.2%) |
|  | 4 | 1/283 (0.4%) |
|  | Missing | 86/283 (30.4%) |
| Alopecia | 1 | 60/249 (24.1%) |
|  | 2 | 114/249 (45.8%) |
|  | Missing | 75/249 (30.1%) |
| Infection | 2 | 49/132 (37.1%) |
|  | 3 | 44/132 (33.3%) |
|  | 4 | 6/132 (4.5%) |
|  | Missing | 33/132 (25.0%) |
| Low white cell count | 1 | 16/97 (16.5%) |
|  | 2 | 24/97 (24.7%) |
|  | 3 | 11/97 (11.3%) |
|  | 4 | 11/97 (11.3%) |
|  | Missing | 35/97 (36.1%) |
| Nausea | 1 | 84/167 (50%) |
|  | 2 | 31/167 (19%) |
|  | 3 | 3/167 (2%) |
|  | Missing | 49/167 (29%) |
| Thrombocytopenia | 1 | 17/29 (59%) |
|  | 2 | 4/29 (14%) |
|  | Missing | 8/29 (28%) |

**Supplementary Table 8 -** **Mean scores and 95% confidence intervals (CIs) adjusted for baseline score for the EQ-5D-5L scale at each timepoint in chemotherapy versus no chemotherapy cohorts.**

| Domain | Time point | | Chemotherapy | No Chemotherapy | Adjusted Mean Difference (95% CI) | P-value |
| --- | --- | --- | --- | --- | --- | --- |
| Score | Baseline | n | 335 | 980 | - | - |
|  |  | Mean (SD) | 0.8769 (0.1327) | 0.8693 (0.1489) |  |  |
|  | 6 weeks | n | 299 | 807 | 0.0130 (-0.0030, 0.0289)  -0.0028 (-0.0231, 0.0175) | 0.112 |
|  |  | Mean (SD) | 0.8409 (0.1414) | 0.8242 (0.1572) |  |  |
|  | 6 months | n | 279 | 760 | -0.0013 (-0.0228, 0.0201)  0.0114 (-0.0143, 0.0372) | 0.789 |
|  |  | Mean (SD) | 0.8247 (0.1767) | 0.8241 (0.1721) |  |  |
|  | 12 months | n | 261 | 659 | 0.0001 (-0.0286, 0.0288)  0.0130 (-0.0030, 0.0289) | 0.903 |
|  |  | Mean (SD) | 0.8274 (0.1639) | 0.8185 (0.1753) |  |  |
|  | 18 months | n | 224 | 556 | -0.0028 (-0.0231, 0.0175)  -0.0013 (-0.0228, 0.0201) | 0.384 |
|  |  | Mean (SD) | 0.8186 (0.1937) | 0.8088 (0.1910) |  |  |
|  | 24 months | n | 185 | 474 | 0.0114 (-0.0143, 0.0372) | 0.994 |
|  |  | Mean (SD) | 0.8013 (0.1787) | 0.8020 (0.1968) |  |  |
| VAS | Baseline | n | 324 | 951 | - | - |
|  |  | Mean (SD) | 78.9 (16.3) | 76.9 (16.6) |  |  |
|  | 6 weeks | n | 296 | 793 | -1.44 (-3.52, 0.64) | 0.176 |
|  |  | Mean (SD) | 74.5 (17.4) | 74.9 (17.0) |  |  |
|  | 6 months | n | 280 | 757 | -6.57 (-8.74, -4.40) | <0.001 |
|  |  | Mean (SD) | 70.1 (18.0) | 75.3 (16.7) |  |  |
|  | 12 months | n | 256 | 656 | 0.72 (-1.60, 3.05) | 0.541 |
|  |  | Mean (SD) | 76.7 (15.8) | 74.8 (17.6) |  |  |
|  | 18 months | n | 230 | 549 | 0.92 (-1.78, 3.63) | 0.503 |
|  |  | Mean (SD) | 74.9 (17.6) | 73.6 (18.3) |  |  |
|  | 24 months | n | 185 | 479 | 1.48 (-1.41, 4.36) | 0.315 |
|  |  | Mean (SD) | 74.7 (16.3) | 72.7 (18.2) |  |  |

**Supplementary figures**

**Supplementary Figure 1**

Logistic regression was used to calculate propensity scores for treatment allocation. The covariates included in the model were measures of functionality (ADL, IADL, MMSE, ECOG), nutritional status (nutrition (abridged PG-SGA), comorbidities (CCI, number of medications) and age. The models were built sequentially as follows:

1. Using scores on their original scale and included patients in whom all scores were completed.
2. Using the categorised versions of the functionality, nutrition and comorbidity again for patients with all scores; age was retained as a continuous term.
3. As ii) including additional patients that could be classified but for whom the score itself was incomplete.

i) and ii) allowed a comparison of models based on categorised and uncategorised scores in order to establish how much information was lost by the categorisations; these were fitted the same participants. Models ii) and iii) differed in the number of individuals available for analysis, since completed inventories could be categorised for some participants. To illustrate taking the IADL, if seven of the eight questions were answered the total score is unknown, but the risk category could be assigned as “high risk” if the answers included two or more limitations. Models i) and ii) performed similarly as demonstrated by the AIC and c-statistics, meaning the simpler categorical model was adequate for creating the propensity scores.

The propensity scores were then used to match chemotherapy patients to patients who did not receive chemotherapy up to a 1:3 ratio. A calliper of 0.25 times the standard deviation of the propensity scores was used to ensure participants were closely matched. This ratio and calliper were chosen based on an examination of the propensity score overlaps for several combinations of ratios and callipers. The figure below shows the overlap in the propensity score for matched sets with different callipers and ratios. Supplementary Table 5 shows the balance achieved in the final matched dataset.

Propensity score overlap and number of observations for matched groups with differing ratios (y-axis) and callipers (x-axis): chemotherapy vs no chemotherapy.


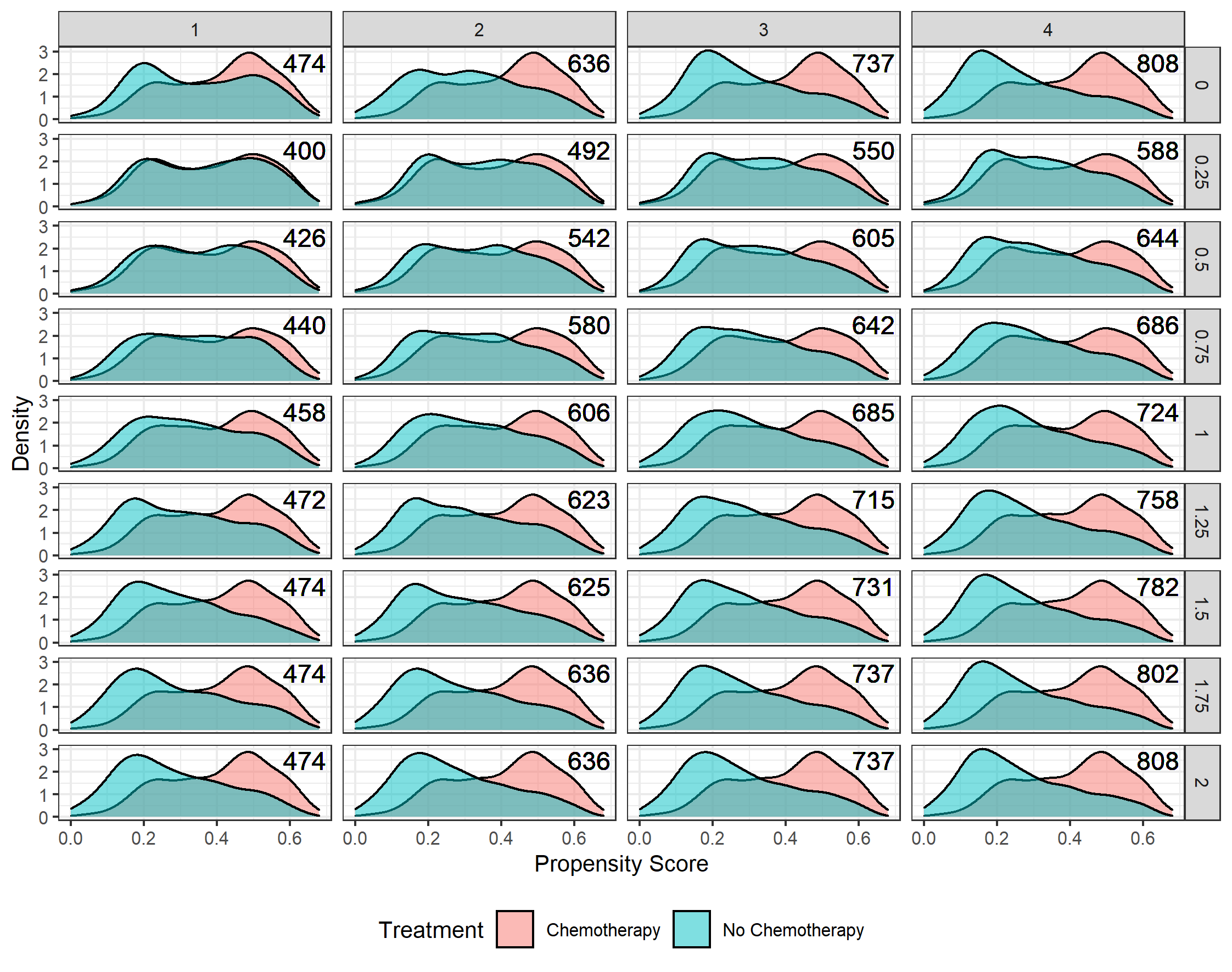


**Supplementary Figure 2 - Kaplan-Meier plots for overall survival (8a) and breast cancer specific survival (8b) for HER2-positive and oestrogen receptor (ER)-negative patients by use of chemotherapy in unmatched population.**

**8a.**
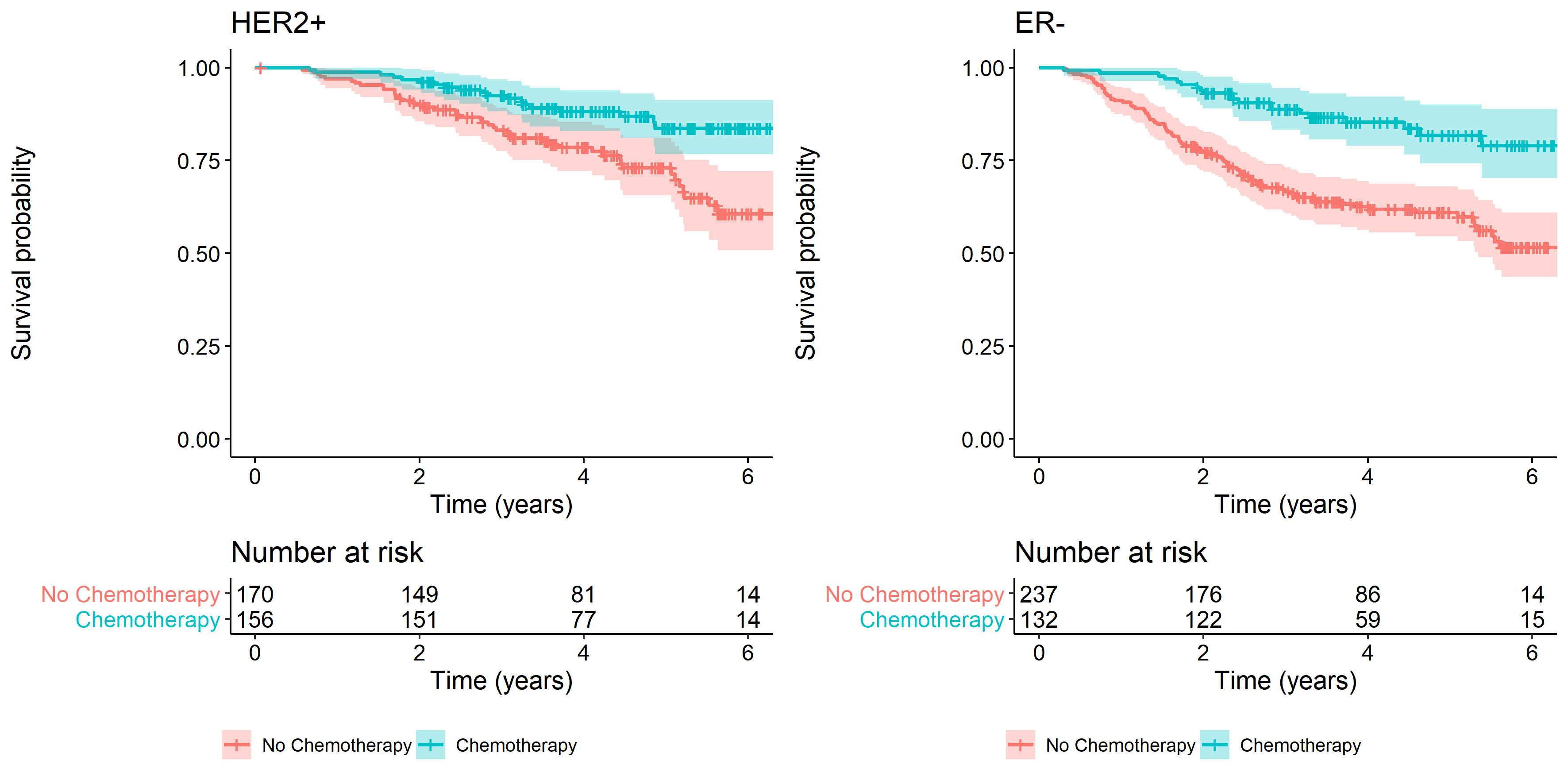


**8b.**


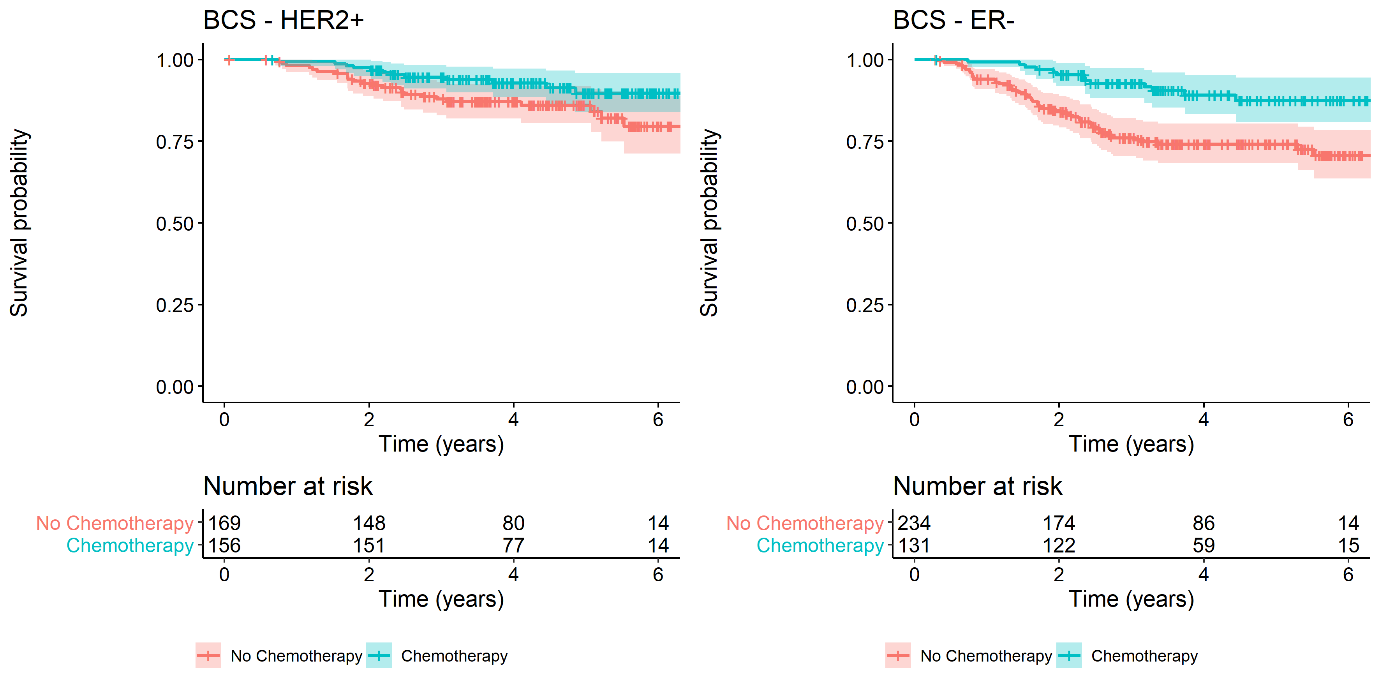


**Supplementary Figure 3 – Mean (95% CI) scores over time points for the chemotherapy versus no chemotherapy population measured on the EQ-5D-5L scale.^*^**


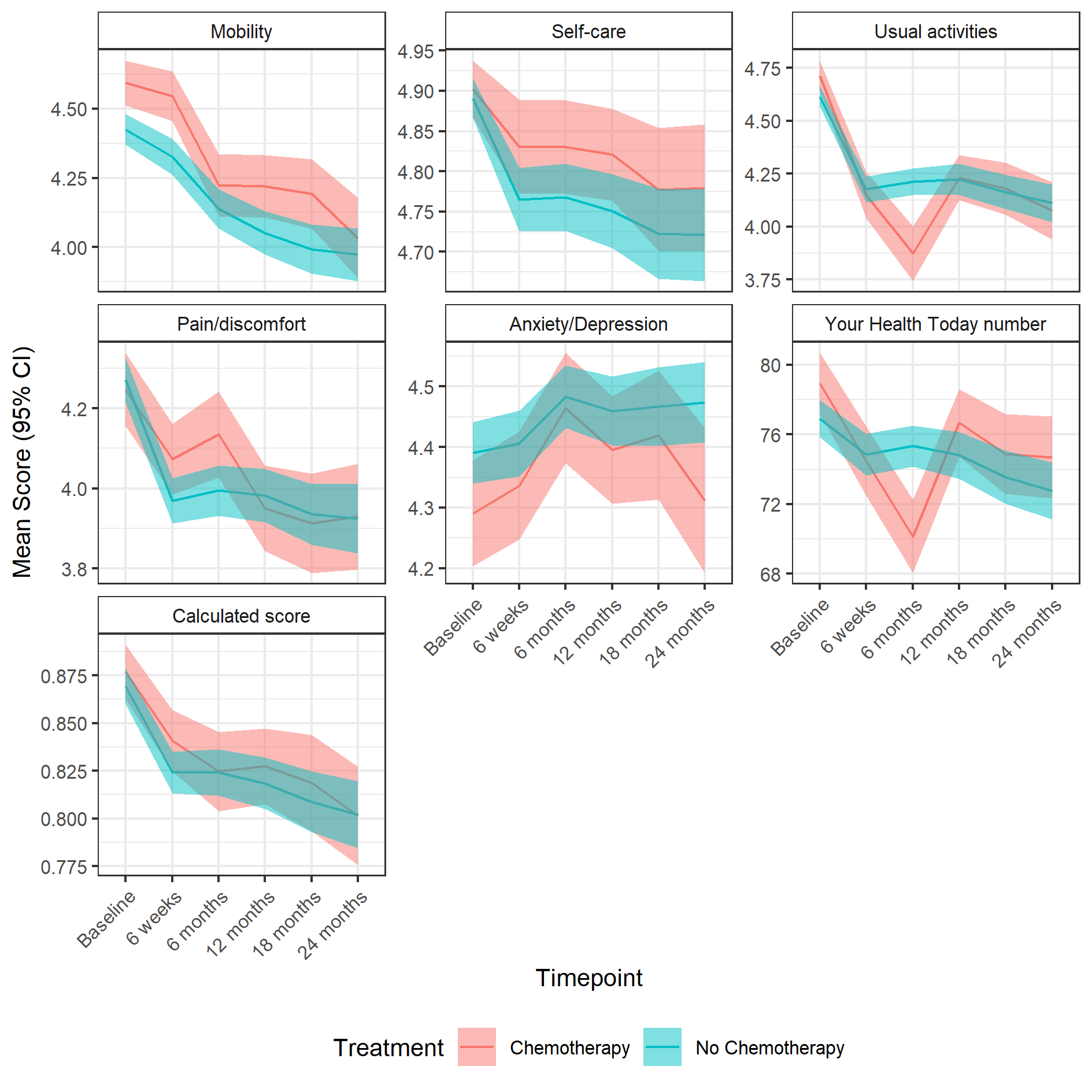


^*^the calculated score is a single summary number (index value) which reflects the health state in the context of the preferences of the general population of a country/region and is derived by applying a formula attaching weights to each of the levels in each dimension as per the EQ-5D-5L User Guide.

**Supplementary Figure 4 – Mean (95% CI) EQ-5D-5L usual activities score over timepoints for the matched chemotherapy vs no chemotherapy population.**


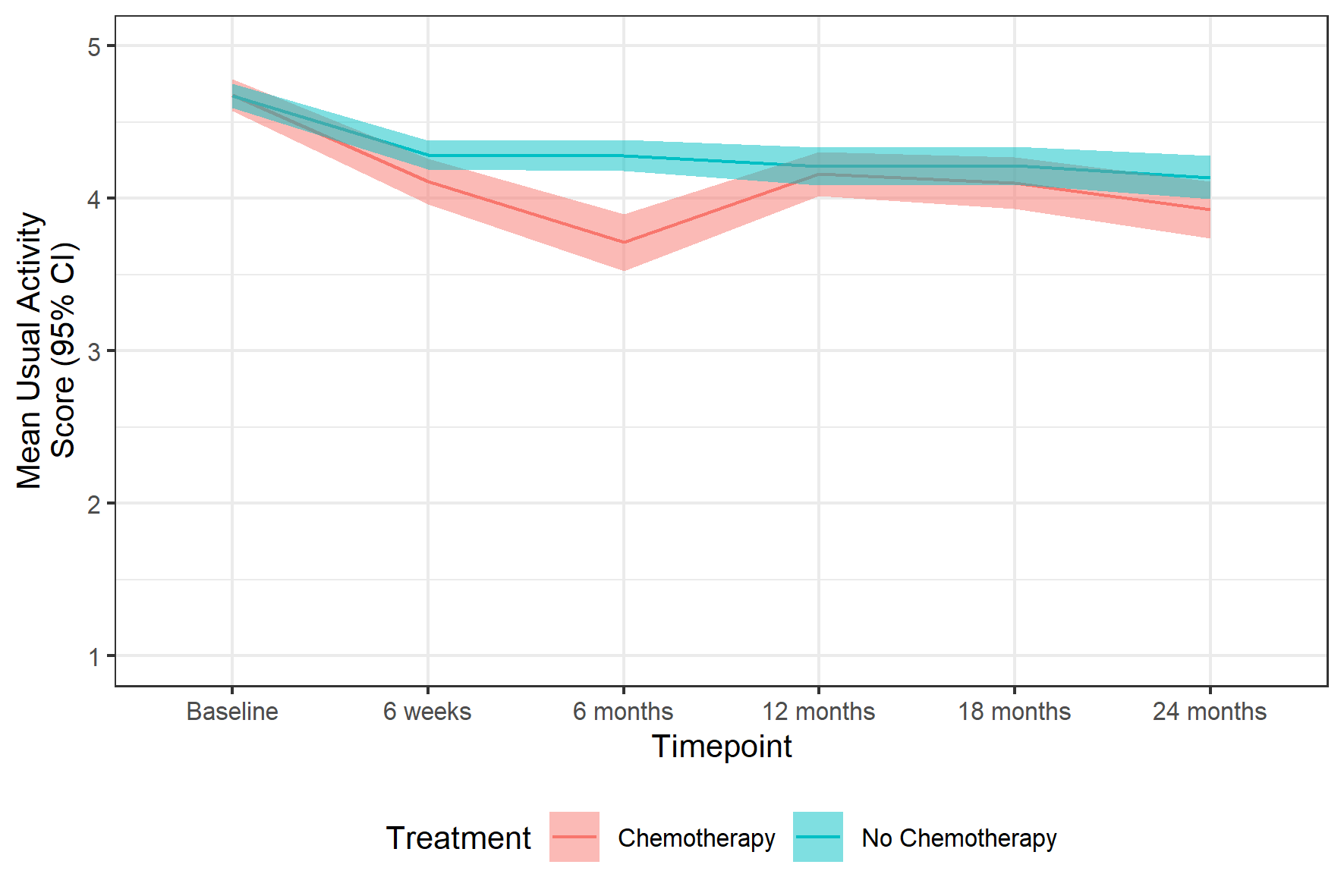

Supplement: Supplementary file 1 — Supplemental materials [file 41416_2021_1388_MOESM1_ESM.docx]
